# Proteasome inhibitor MG132 inhibits the proliferation and promotes the cisplatin-induced apoptosis of human esophageal squamous cell carcinoma cells

**DOI:** 10.3892/ijmm.2014.1678

**Published:** 2014-02-27

**Authors:** LIFENG DANG, FENGBIAO WEN, YANG YANG, DONGLEI LIU, KAI WU, YU QI, XIANGNAN LI, JIA ZHAO, DENGYAN ZHU, CHUNYANG ZHANG, SONG ZHAO

**Affiliations:** 1 Physical Examination Centre, First Affiliated Hospital, Zhengzhou University, Zhengzhou, Henan 450052, P.R. China; 2 Department of Thoracic Surgery, First Affiliated Hospital, Zhengzhou University, Zhengzhou, Henan 450052, P.R. China; 3 Open Key Clinical Medical Experimental Laboratory Institute of Henan Province, Zhengzhou, Henan 450052, P.R. China; 4 Institute of Molecular Cancer Surgery of Zhengzhou University, Zhengzhou, Henan 450052, P.R. China; 5 Key Thoracic Tumour Experimental Laboratory of Zhengzhou, Zhengzhou, Henan 450052, P.R. China

**Keywords:** proteosome inhibitor, MG132, apoptosis, esophageal squamous cell carcinoma

## Abstract

Comprehensive treatment based on chemotherapy is regarded as the first-line treatment for patients with unresectable or metastatic esophageal squamous cell carcinoma (ESCC). However, chemoresistance is common among patients with ESCC. Therefore, there is a need to explore new therapeutic strategies or adjuvant drugs. One promising possibility is to use dietary agents that can increase tumor cell sensitivity to drugs. In this study, we initially investigated the antitumor activity of proteasome inhibitor MG132 *in vitro* and *in vivo*. Effects of MG132 on the enhancment of the anticancer functions of cisplatin were then investigated in human esophageal cancer EC9706 cells in relation to apoptosis and cell signaling events. Exposure of cells to MG132 resulted in a marked decrease in cell viability in a dose- and time-dependent manner. Administration of MG132 markedly inhibited tumor growth in the EC9706 xenograft model. MG132 significantly enhanced cisplatin-induced apoptosis in association with the activation of caspase-3 and -8. These events were accompanied by the downregulation of NF-κB, which plays a key role in cell apoptosis. Taken together, these findings demonstrate a novel mechanism by which proteasome inhibitor MG132 potentiates cisplatin-induced apoptosis in human ESCC and inhibitory activity of tumor growth of the EC9706 xenograft model.

## Introduction

Esophageal squamous cell carcinoma (ESCC) is one of the most lethal types of cancer worldwide owing to its extremely aggressive nature and poor survival rate (1). The early diagnosis of esophageal cancer is difficult, therefore, once diagnosed, most of the patients lack the optimal time required for surgical treatment. Comprehensive treatment based on chemotherapy is regarded as the first-line treatment for patients with unresectable or metastatic ESCC (2). However, tumor cells often develop resistance to chemotherapeutic agents during the treatment (3). For this reason, it is crucial to identify new therapeutic strategies or adjuvant drugs. A promising possibility is to use dietary agents that can increase tumor cell sensitivity to drugs (4).

MG132, which acts as a blocker in ubiquitin-proteasome pathway, is involved in >80% of intracellular protein degradation (5). Recently, it was shown that the intrinsic resistance to apoptosis is an important mechanism by which cancer cells can escape therapeutic control (6). Stoll *et al* provided convincing evidence to suggest an essential role for MG132 in apoptosis (7). Findings of previous studies have indicated that MG132 enhances cisplatin-induced apoptosis in various types of tumor cells (8–10). However, the molecular mechanisms of MG132-related lethality in esophageal squamous cancer cells are not fully defined.

In this study, we initially investigated the antitumor activity of proteasome inhibitor MG132 *in vitro* and *in vivo*. Effects of MG132 on enhancing the anticancer functions of cisplatin were then investigated in human esophageal cancer EC9706 cells in relation to apoptosis and cell signaling events.

## Materials and methods

### Cell culture

EC9706, EC109, EC1 and TE-1 cells were obtained from the Open Key Clinical Medical Experimental Laboratory Institute of Henan Province (Henan, China). The cells were cultured in RPMI-1640 medium (Life Technologies, Grand Island, NY, USA) supplemented with 10% FBS (Sijiqing Biological Engineering Materials Co., Ltd., Hangzhou, China), 100 U/ml penicillin and 100 μg/ml streptomycin in a humidified atmosphere (5% CO_2_) at 37ºC with medium changes every two days. Cells in the logarithmic growth phase were used in this study.

### Cell viability assay

Cell proliferation was assessed using a cell counting kit (CCK-8) (Dojindo Laboratories, Kumamoto, Japan), following the manufacturer’s instructions. Cells (5×10^5^/ml) were seeded in 96-well plates with 200 μl in each well, and added to the culture medium containing agents of different concentrations (1, 5 and 10 μM) or control PBS with 100 μl in each well, each concentration for parallel 4-wells after adherence. Following culture, the medium was removed and 100 μl fresh medium containing 10 μl CCK-8 was added to each well. The cells were then incubated at 37ºC for 4 h. The optical density values were determined in at least triplicate against a reagent blank at a test wavelength of 490 nm and a reference wavelength of 630 nm. Cell viability was calculated as a percentage as follows: (A_treated_/A_control_) ×100.

### Xenograft tumor growth

Female athymic nude mice (5- to 6-weeks old) were inoculated intraperitoneally (i.p.) with 7×10^6^ EC9706 cells, after which mice received injections with vehicle or MG132 (10 mg/kg, i.p.) for 25 days starting 5 days after the injection of EC9706 cells. Twenty nude mice were randomly divided into two groups (n=10 per group). Mice were fed with antioxidant-free AIN-76A special diet for a week before starting the experiment.

### Apoptosis analysis

Adherent cells were digested into suspension of single cells with EDTA-free trypsin and then washed twice with cold PBS. To assess apoptosis, EC9706 cells were stained by Annexin V-FITC/PI using an Annexin V-FITC Apoptosis Detection kit according to the manufacturer’s instructions. The presence of fluorescent images was immediately verified microscopically. Flow cytometry was used to quantify the level of cell apoptosis.

### Western blotting

Cells were collected and lysed for 20 min in cold buffer. Cell extracts were collected and centrifuged at 12,000 rpm for 5 min. Proteins (20 μg) from whole cell lysates were boiled for 5 min in SDS buffer, resolved by 12% SDS-PAGE, then electrotransferred to nitrocellulose membranes by semi-dry transfer, blocked overnight at 4ºC and incubated for 1 h with primary antibodies at the recommended concentrations for caspase-8, caspase-3, NF-κB and β-actin. The membranes were then incubated with horseradish peroxidase-conjugated secondary antibodies (anti-rabbit or anti-mouse IgG). Blots were developed using enhanced chemiluminescence and visualized on Kodak X-omat LS film. Densitometry was performed with Kodak ID image analyses software.

### Statistical analysis

Experiments were performed in triplicate and quantitative results were expressed as the mean ± standard deviation (SD). Statistical analysis was performed using the statistical program SPSS 13.0. Data were compared using standard ANOVA methodology for repeated measurement, followed by the Student’s t-test. P<0.05 was considered to indicate statistical significance.

## Results

### Proteasome inhibitor MG132 suppressed the proliferation of EC9706 cells in a dose- and time-dependent manner

**Figure 1:**
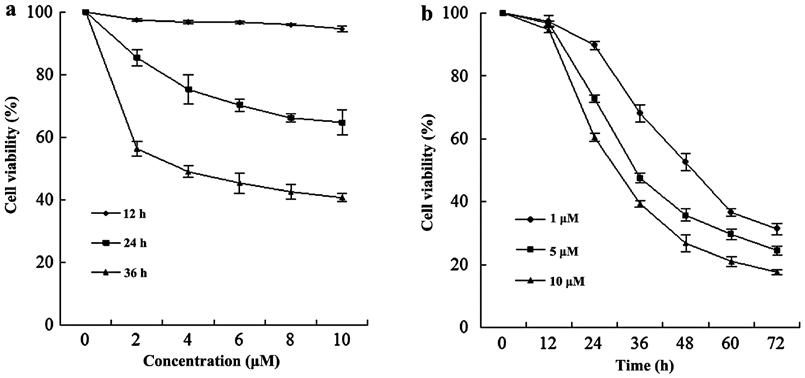
Proteasome inhibitor MG132 significantly decreased the cell viability in EC9706 cells in a dose- and time-dependent manner. (a) EC9706 cells were treated with or without various concentrations of MG132 as indicated for 12, 24 and 36 h. (b) EC9706 cells were treated with 1, 5 and 10 μM for 12, 24, 36, 48, 60 and 72 h.

A dose-dependent study of EC9706 cells exposed to various concentrations of MG132 for 12, 24 and 36 h is shown in Fig. 1a; the modest degrees of growth inhibition were noted at a concentration of 2 μM, which increased substantially at a concentration of 4 μM. These events were significantly increased at a concentration of 10 μM. A time-course study of cells exposed to MG132 revealed a significant increase in cell viability as early as 24 h, and reached near-maximal levels after 60 h (Fig. 1b).

### MG132 had similar antitumor effects on multiple human esophageal squamous cancer cells

**Figure 2:**
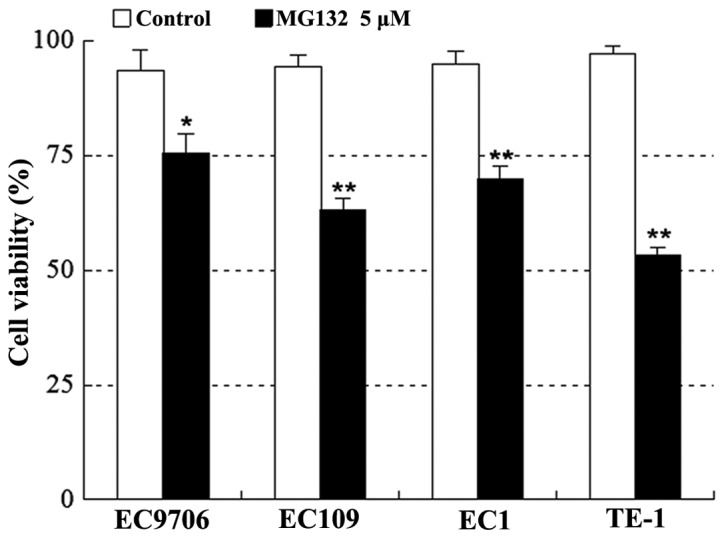
Proteasome inhibitor MG132 had similar effects on cell viability in other human esophageal squamous cancer cell lines as well as EC9706 cells. EC9706, EC109, EC1 and TE-1 cells were treated with or without 5 μM MG132 for 24 h. The absorbance (A) obtained from CCK-8 assays is the mean ± standard deviation (SD) for three separate experiments. Absorbance for cells treated with MG132 were significantly reduced compared with that obtained for the control as assessed using the Student’s t-test (^*^P<0.05, ^**^P<0.01 vs. the control group).

Various human esophageal squamous cancer cells, including EC9706, EC109, EC1 and TE-1, were exposed to 5 μM MG132 for 24 h, after which apoptosis was determined by CCK-8 assay. As shown in Fig. 2, treatment with MG132 resulted in a marked decrease in cell viability in the four cell lines.

### MG132 inhibited tumor growth in EC9706 xenograft animal model

**Figure 3:**
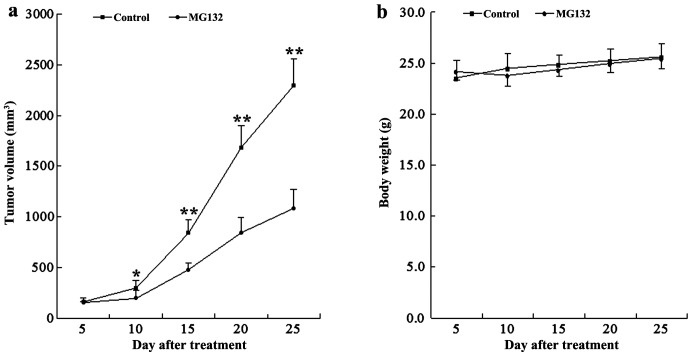
MG132 suppressed the growth of esophageal cancer xenografts. Twenty nude mice were randomly divided into two groups (n=10 per group) for treatment with MG132 [10 mg/kg, intraperitoneally (i.p.) daily] or with vehicle control solvent. (a) Average tumor volume in vehicle control mice and mice treated with MG132. Tumor volumes of xenograft mice treated with MG132 were significantly decreased compared with vehicle control assessed using the Student’s t-test (^*^P<0.05, ^**^P<0.01 vs. the control group). (b) There was no statistically significant difference in body weight between vehicle control and MG132 regimen (P>0.05).

To assess whether our *in vitro* observations could be translated into an animal model system, athymic nude mice were inoculated i.p. with EC9706 cells, after which mice received injections with vehicle or MG132 (10 mg/kg, i.p.) for 25 days starting 5 days after the injection. As shown in Fig. 3a, treatment with MG132 resulted in a modest, but significant suppression of tumor growth 10 days following drug exposure (P<0.05 vs. vehicle control). These events became more apparent 15, 20 and 25 days after drug exposure (P<0.01 between MG132 treatment and vehicle control). By contrast, no statistically significant change in body weight was noted when compared with the vehicle control and MG132 regimen (Fig. 3b). Moreover, the mice of MG132 group did not exhibit any other signs of toxicity such as agitation, impaired movement, posture, indigestion, diarrhea or areas of redness. These results indicated that MG132 administration significantly inhibited tumor growth of the EC9706 xenograft without causing toxicity to the mice.

### Cell viability and morphological changes of EC9706 cells

**Figure 4:**
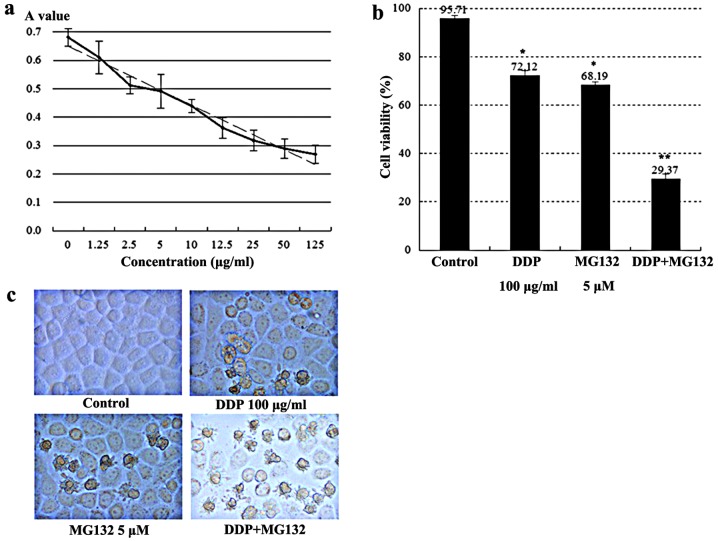
The inhibitory effect on EC9706 cells after 24, 48 and 72 h measured by CCK-8 assay. (a) EC9706 cells were treated with different concentrations (0–125 μg/ml) of DDP for 24 h and the cell viability was determined by a CCK-8 assay. (b) The survival rate of EC9706 cells with 100 μg/ml DDP, 5 μM MG132 and a combination of the two. (^*^P<0.05 vs. the control group, ^**^P<0.01 vs. the DDP and MG132 groups). (c) Morphological changes of EC9706 cells after 24 h of treatment with different drugs. Images captured under a microscope; magnification, ×400.

Exposure of cells to a series of concentrations of cisplatin for 24 h resulted in a significant dose-inhibition effect between the different groups (P<0.05). There was a linear relationship between cisplatin concentration and the A value (Fig. 4a), where the correlation coefficient was r=−0.023 (P<0.001) and the linear regression equation was A value=0.735–0.0018 × cisplatin concentration (μg/ml). The proliferation inhibitory rate of cisplatin on EC9706 cells was 25% when the drug concentration was 100 μg/ml. Then, 100 μg/ml was selected as cisplatin concentration in the follow-up studies.

Addition of 5 μM MG132 for 24 h resulted in a marked decrease in cell viability in the combined group as compared with the individual agents (P<0.01) (Fig. 4b). The results obtained suggested that the combined use of DDP and MG132 had stronger cytotoxicity than the single agent.

Normal EC9706 cells were polygonal in shape with a high refractive index and large cell volumes. The cells were stretched tightly and adhered to the wall, and had a cobblestone-like appearance. In the MG132 5 μM and/or DDP 100 μg/ml group, some cells decreased into round shapes, with a reduced refractive index. The cells were detached from the wall and floated in culture medium. A significant increase was observed in these events in the combined group of MG132 (5 μM) and DDP (100 μg/ml) (Fig. 4c).

### Effect of DDP and MG132 used individually or in combination on EC9706 cell apoptosis

**Figure 5:**
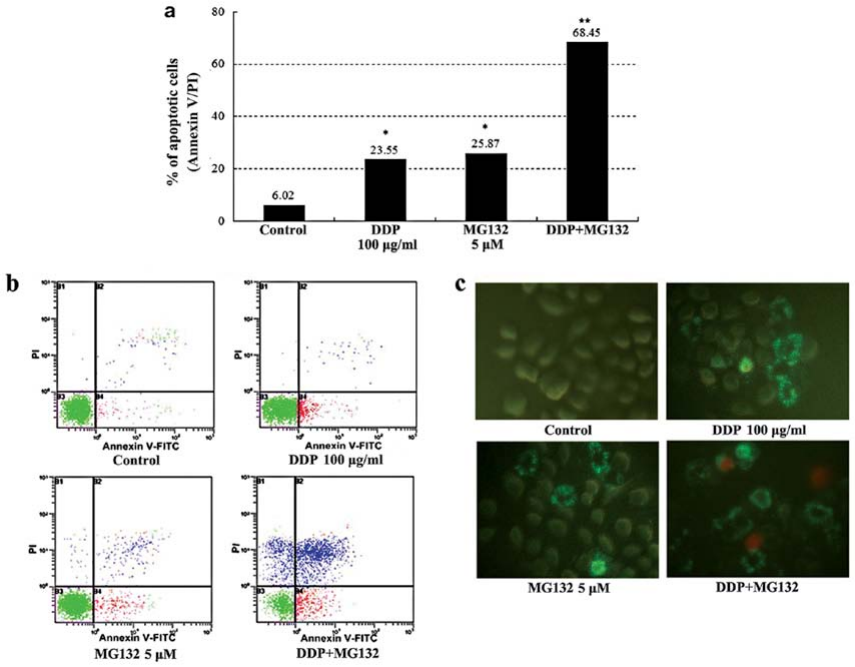
Apoptosis in EC9706 cells treated with different drugs after 24 h. (a) The four groups (blank control group, 100 μg/ml DDP group, 5 μM MG132 group, 100 μg/ml DDP and 5 μM MG132 group) were incubated for 24 h, cells were stained with FITC-conjugated Annexin V and PI, followed by flow cytometric analysis. The percentages of apoptotic cells were obtained from three independent experiments (^*^P<0.05 vs. the control group, ^**^P<0.01 vs. DDP and MG132 group). (b) Early apoptosis was defined by Annexin V^+^/PI^-^ staining (B4), and late apoptosis was defined by Annexin V^+^/PI^+^ staining (B2). (c) Cells of the four groups were stained with Annexin V-FITC and PI. Images captured under a microscope; magnification, ×400.

Counts of apoptotic cells detected by flow cytometry are shown in Fig. 5a and b: The apoptotic percentage of cells for the DDP + MG132 group was much higher than that in the blank control and individual groups (P<0.01). The addition of MG132, increased the cisplatin-induced apoptosis rate from 23 to 68%. Annexin V-FITC and PI staining was used to estimate the extent of cell apoptosis. Observed under a fluorescent microscope, Annexin V-FITC^+^ cells appeared as bright apple green on the cell membrane, whereas PI^+^ cells had different intensities of yellow red throughout the cytoplasm. Annexin V-FITC^+^ cells were rarely observed in the control group, while many positive cells were visible in the MG132 group, DDP group and, particularly the DDP + MG132 group (Fig. 5c). The results obtained suggested that MG132 is able to enhance cisplatin-induced apoptosis in esophageal cancer cells.

### Expression of NF-κB, caspase-8 and -3

**Figure 6:**
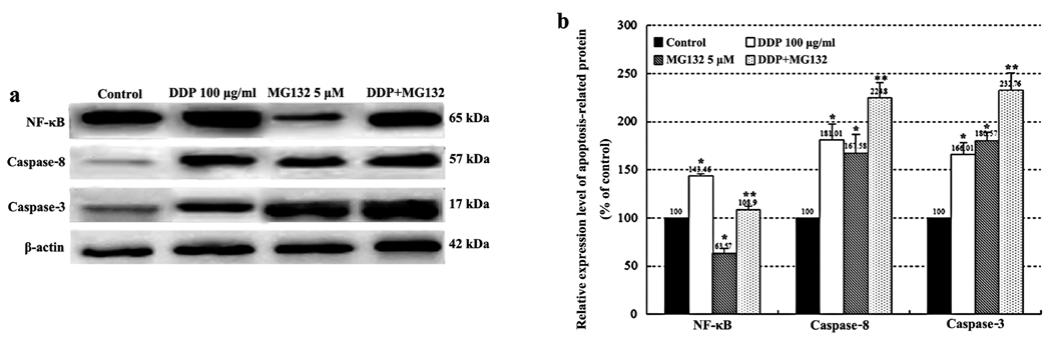
The expression of NF-κB, caspase-8 and -3 determined by western blotting. The mean ± standard deviation (SD) of the results were obtained from three independent experiments. (^*^P<0.05 vs. the control group, ^**^P<0.01 vs. the DDP and MG132 groups).

As shown in Fig. 6, activation of caspase-8 and -3, and NF-κB was determined by western blotting after 24 h of DDP treatment. The expression of caspase-8, -3 and NF-κB was upregulated in the DDP group as compared with the control group. In addition, a combination of DDP and MG132 treatment increased the expression levels of caspase-8 and -3 when compared with DDP or MG132 alone (P<0.05). Conversely, MG132 partially counteracted the upregulation of NF-κB in the combined group compared with the DDP group (P<0.01).

## Discussion

Comprehensive treatment based on chemotherapy is regarded as the first-line treatment for patients with unresectable or metastatic ESCC (11). However, chemoresistance is common among patients with ESCC (12–14). Therefore, it is crucial to identify new therapeutic strategies or adjuvant drugs. A promising possibility is to use dietary agents that potentially increase tumor cell sensitivity to drugs.

As a natural triterpene proteasome inhibitor extracted from a Chinese medicinal plant, MG132 has been investigated in several cancer cells in relation to apoptosis and cell signaling events (15,16). In the present study, we have shown that MG132 suppressed the growth of esophageal cancer cells in culture as well as in the animal model. The experimental results *in vitro* demonstrated that MG132 inhibited the proliferation in EC9706 cells in a dose- and time-dependent manner, with the concentration increasing from 0 to 10 μM and the survival rate decreasing from 100 to 18.43% after 36 h (Fig. 1). MG132 also markedly induced cell death in multiple human esophageal cancer cell types, including EC9706, EC109, EC1 and TE-1 cell lines. The results from *in vivo* studies about xenograft model showed that MG132 exhibited significant inhibitory effects on the growth of esophageal cancer xenograft.

In order to justify whether or not the application of MG132 could improve the sensitivity to chemotherapy drugs in esophageal cancer cells, the cells were divided into the blank control, 100 μg/ml DDP, 5 μM MG132, 100 μg/ml DDP and 5 μM MG132 groups. To elucidate the potential mechanism underlying the tumor-suppressive function of combined DDP and MG132 treatment, we examined the proliferation and apoptosis of esophageal cancer EC9706 cells. Exposure of cells to cisplatin (DDP) combined with MG132 resulted in a marked increase in the cell cytotoxicity of esophageal cancer cells as compared with the single agent (Fig. 4b). This result was confirmed by the Annexin V-FITC apoptosis detection assay, which showed that the combined DDP and MG132 treatment induced more apoptosis in tumor cells than in DDP treatment alone (68.45±2.58 vs. 23.5±1.23%; P<0.01, Fig. 5). Our findings provide convincing evidence that the combined treatment of DDP and proteasome inhibitor MG132 exerts a synergistic apoptotic effect as compared with each agent alone.

There are two pathways in apoptosis: the cell surface death receptor pathway and the mitochondria-initiated pathway (17,18). In the cell surface receptor pathway, activation of caspases following their recruitment to the death-inducing signaling complex is the critical event that transmits the death signal (19–21). Apoptosis requires a cascade of complex biochemical events that are performed with the participation of a family of cysteine proteases known as caspases (22). Specifically, caspase-8 and -3 have been viewed as the essential regulators in apoptosis cascade (23,24). NF-κB is a ubiquitous transcription factor that plays a key role in basic processes such as the regulation of cell proliferation and apoptosis (25–27). To identify the mechanism by which proteasome inhibitor MG132 potentiates cisplatin-induced apoptosis in human esophageal squamous cancer cells, we investigated changes of the expression levels of caspase-8, -3 and NF-κB after treatment with DDP and MG132 individually or in combination. The results of our study suggest that MG132 significantly enhanced cisplatin-induced apoptosis in association with the activation of caspase-3 and -8. These events were accompanied by the downregulation of the NF-κB pathway which plays a key role in cell apoptosis (Fig. 6). Activation of the NF-κB pathway resulting in reduced susceptibility to cisplatin in esophageal cancer cells may play an important role in drug resistance induced by cisplatin. MG132 can significantly enhance the sensitivity of esophageal cancer cells to cisplatin and effectively improve the rate of cell apoptosis by inhibiting the activation of NF-κB, potentiating the expression levels of apoptosis-related protein caspase-8 and -3.

In summary, these findings indicate that proteasome inhibitor MG132 may promote cisplatin-induced apoptosis by inhibiting the activation of NF-κB and upregulating the expression levels of caspase-3 and -8. MG132 may be used alone or in combination with other therapeutic agents to treat ESCC. However, our findings require further investigations to clarify the molecular mechanisms underlying the results of the present study. Findings of this study have proivded a novel and promising therapeutic strategy which is likely to benefit the clinical treatment of patients with ESCC.

## References

[1] van Hagen P, Hulshof MC, van Lanschot JJ (2012). Preoperative chemoradiotherapy for esophageal or junctional cancer. N Engl J Med.

[2] Tomblyn MB, Goldman BH, Thomas CR (2012). Cetuximab plus cisplatin, irinotecan, and thoracic radiotherapy as definitive treatment for locally advanced, unresectable esophageal cancer: a phase-II study of the SWOG (S0414). J Thorac Oncol.

[3] Murtaza M, Dawson SJ, Tsui DW (2013). Non-invasive analysis of acquired resistance to cancer therapy by sequencing of plasma DNA. Nature.

[4] Gao D, Hu J, Zhang X, Gao C, Hong J (2013). Effect of hOGG1 over-expression on cisplatin resistance in esophageal squamous carcinoma cells. Cancer Biother Radiopharm.

[5] Khedgikar V, Kushwaha P, Gautam J (2013). Withaferin A: a proteasomal inhibitor promotes healing after injury and exerts anabolic effect on osteoporotic bone. Cell Death Dis.

[6] Li X, Huang T, Jiang G, Gong W, Qian H, Zou C (2013). Proteasome inhibitor MG132 enhances TRAIL-induced apoptosis and inhibits invasion of human osteosarcoma OS732 cells. Biochem Biophys Res Commun.

[7] Stoll SJ, Pitt SC, Chen H (2009). Follicular thyroid cancer cell growth inhibition by proteosome inhibitor MG132. J Surg Res.

[8] Duraj J, Pastorek M, Vitkovska J (2013). Proteasome inhibition leads to altered signaling in the proteome of cisplatin-resistant human ovarian carcinoma cell line. Neoplasma.

[9] Fribley AM, Evenchik B, Zeng Q (2006). Proteasome inhibitor PS-341 induces apoptosis in cisplatin-resistant squamous cell carcinoma cells by induction of Noxa. J Biol Chem.

[10] Fribley A, Zeng Q, Wang CY (2004). Proteasome inhibitor PS-341 induces apoptosis through induction of endoplasmic reticulum stress-reactive oxygen species in head and neck squamous cell carcinoma cells. Mol Cell Biol.

[11] He YF, Ji CS, Hu B (2013). A phase II study of paclitaxel and nedaplatin as front-line chemotherapy in Chinese patients with metastatic esophageal squamous cell carcinoma. World J Gastroenterol.

[12] Kurosu T, Nagao T, Wu N, Oshikawa G, Miura O (2013). Inhibition of the PI3K/Akt/GSK3 pathway downstream of BCR/ABL, Jak2-V617F, or FLT3-ITD downregulates DNA damage-induced Chk1 activation as well as G2/M arrest and prominently enhances induction of apoptosis. PLoS One.

[13] Chen WW, Lin CC, Huang TC, Cheng AL, Yeh KH, Hsu CH (2013). Prognostic factors of metastatic or recurrent esophageal squamous cell carcinoma in patients receiving three-drug combination chemotherapy. Anticancer Res.

[14] Shi Y, Qin R, Wang ZK, Dai GH (2013). Nanoparticle albumin-bound paclitaxel combined with cisplatin as the first-line treatment for metastatic esophageal squamous cell carcinoma. Onco Targets Ther.

[15] Tu Y, Chen C, Pan J, Xu J, Zhou ZG, Wang CY (2012). The Ubiquitin Proteasome Pathway (UPP) in the regulation of cell cycle control and DNA damage repair and its implication in tumorigenesis. Int J Clin Exp Pathol.

[16] Yang H, Landis-Piwowar K, Chan TH, Dou QP (2011). Green tea polyphenols as proteasome inhibitors: implication in chemoprevention. Curr Cancer Drug Targets.

[17] Kandilis AN, Karidis NP, Kouraklis G, Patsouris E, Vasileiou I, Theocharis S (2014). Proteasome inhibitors: possible novel therapeutic strategy for ischemia-reperfusion injury?. Expert Opin Investig Drugs.

[18] Flemming A (2011). Therapeutics: opening the door to a new class of proteasome inhibitors. Nat Rev Cancer.

[19] Chen FZ, Zhao XK (2013). Ubiquitin-proteasome pathway and prostate cancer. Onkologie.

[20] Gu JJ, Hernandez-Ilizaliturri FJ, Kaufman GP (2013). The novel proteasome inhibitor carfilzomib induces cell cycle arrest, apoptosis and potentiates the anti-tumour activity of chemotherapy in rituximab-resistant lymphoma. Br J Haematol.

[21] Hui B, Shi YH, Ding ZB (2012). Proteasome inhibitor interacts synergistically with autophagy inhibitor to suppress proliferation and induce apoptosis in hepatocellular carcinoma. Cancer.

[22] Altmann A, Markert A, Askoxylakis V (2012). Antitumor effects of proteasome inhibition in anaplastic thyroid carcinoma. J Nucl Med.

[23] Rastogi N, Mishra DP (2012). Therapeutic targeting of cancer cell cycle using proteasome inhibitors. Cell Div.

[24] da Cunha FM, Demasi M, Kowaltowski AJ (2011). Aging and calorie restriction modulate yeast redox state, oxidized protein removal, and the ubiquitin-proteasome system. Free Radic Biol Med.

[25] Chitra S, Nalini G, Rajasekhar G (2012). The ubiquitin proteasome system and efficacy of proteasome inhibitors in diseases. Int J Rheum Dis.

[26] de Almagro MC, Vucic D (2012). The inhibitor of apoptosis (IAP) proteins are critical regulators of signaling pathways and targets for anti-cancer therapy. Exp Oncol.

[27] Napetschnig J, Wu H (2013). Molecular basis of NF-κB signaling. Annu Rev Biophys.

